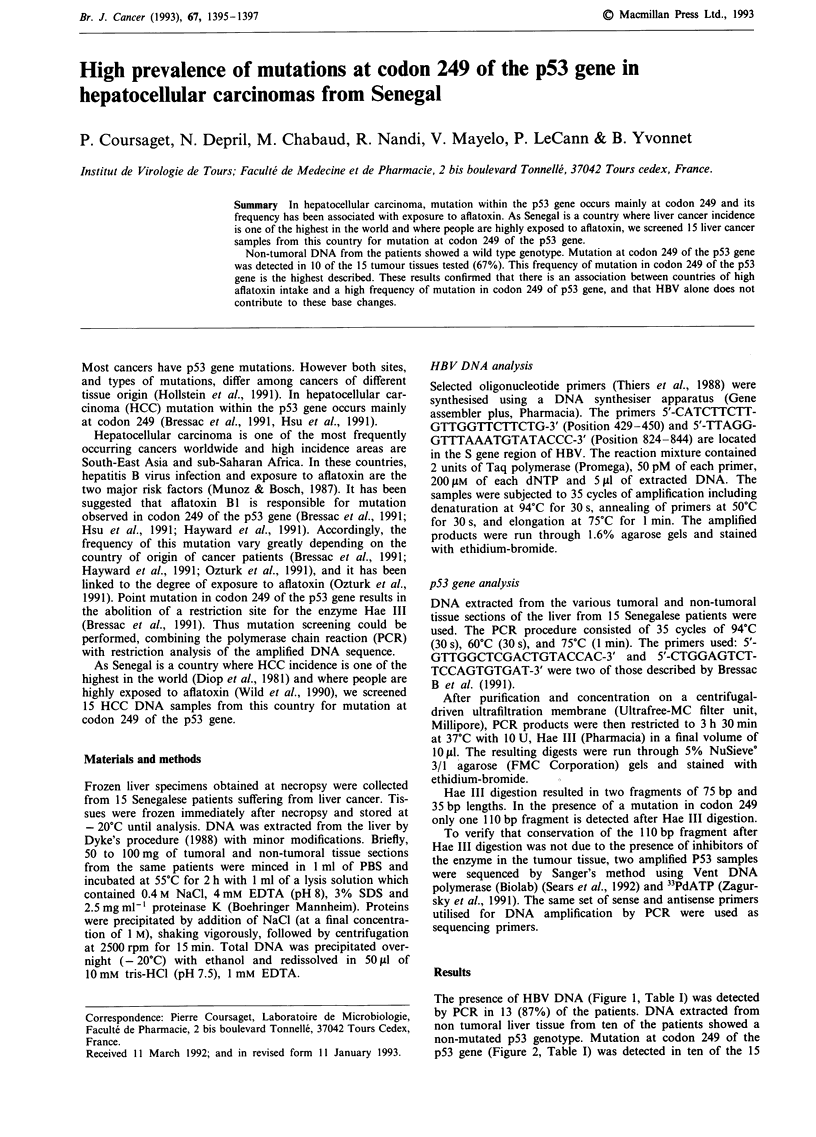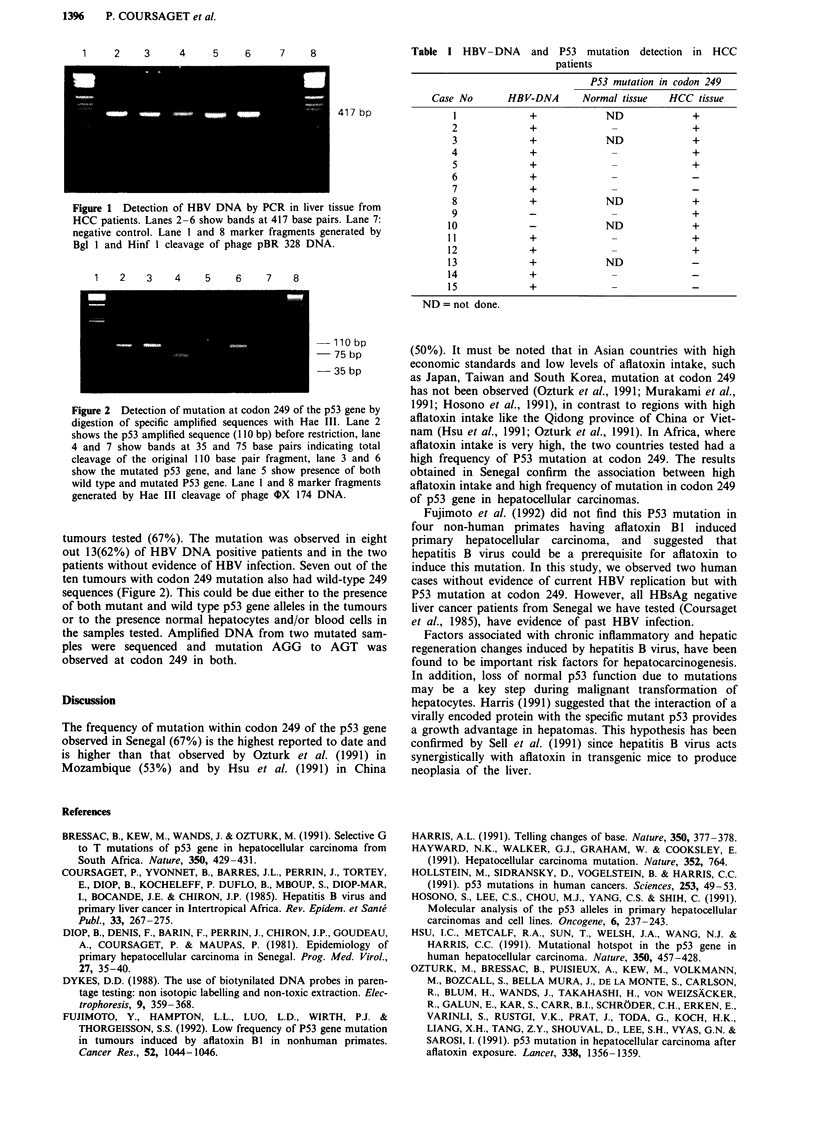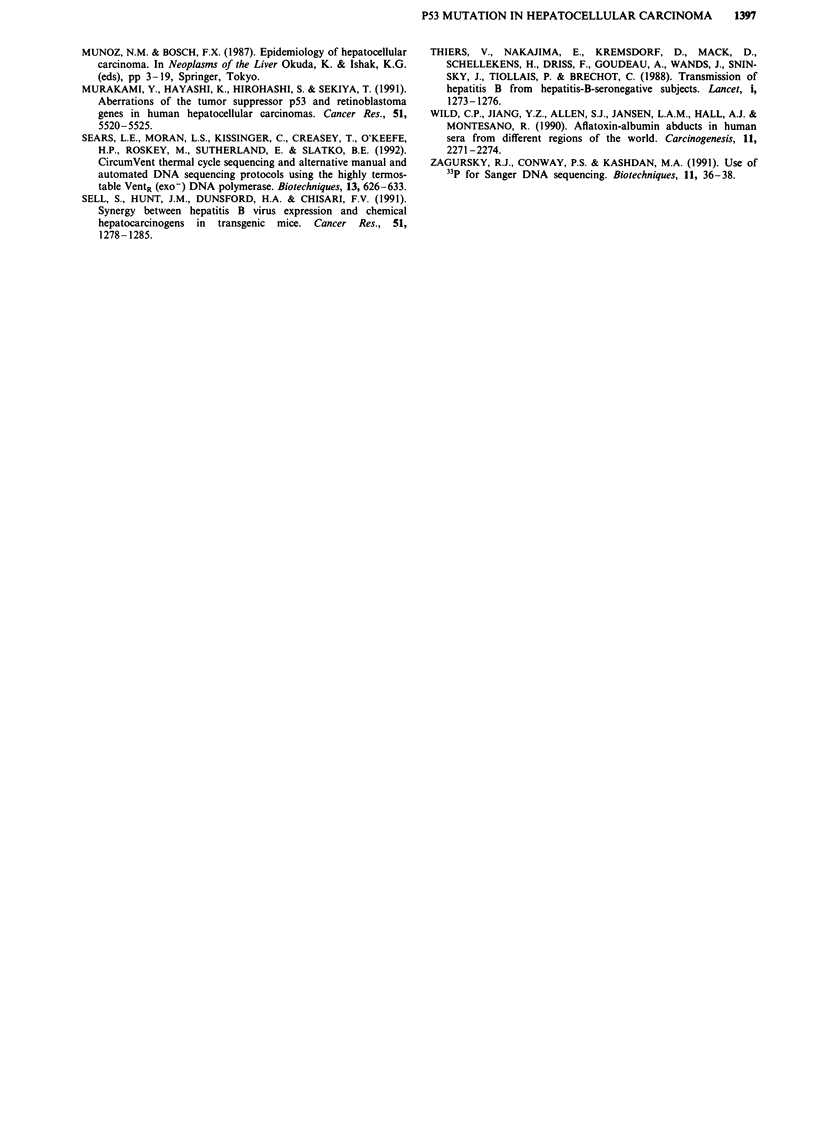# High prevalence of mutations at codon 249 of the p53 gene in hepatocellular carcinomas from Senegal.

**DOI:** 10.1038/bjc.1993.258

**Published:** 1993-06

**Authors:** P. Coursaget, N. Depril, M. Chabaud, R. Nandi, V. Mayelo, P. LeCann, B. Yvonnet

**Affiliations:** Institut de Virologie de Tours, Faculté de Medecine et de Pharmacie, France.

## Abstract

**Images:**


Br. J. Cancer (1993), 67, 1395–1397 © Macmillan Press Ltd., 1993

### High prevalence of mutations at codon 249 of the p53 gene in hepatocellular carcinomas from Senegal

P. Coursaget, N. Depril, M. Chabaud, R. Nandi, V. Mayelo, P. LeCann & B. Yvonnet

*Institut de Virologie de Tours; Faculté de Medecine et de Pharmacie, 2 bis boulevard Tonnellé, 37042 Tours cedex, France.*

**Summary** In hepatocellular carcinoma, mutation within the p53 gene occurs mainly at codon 249 and its frequency has been associated with exposure to aflatoxin. As Senegal is a country where liver cancer incidence is one of the highest in the world and where people are highly exposed to aflatoxin, we screened 15 liver cancer samples from this country for mutation at codon 249 of the p53 gene.

Non-tumoral DNA from the patients showed a wild type genotype. Mutation at codon 249 of the p53 gene was detected in 10 of the 15 tumour tissues tested (67%). This frequency of mutation in codon 249 of the p53 gene is the highest described. These results confirmed that there is an association between countries of high aflatoxin intake and a high frequency of mutation in codon 249 of p53 gene, and that HBV alone does not contribute to these base changes.

Most cancers have p53 gene mutations. However both sites, and types of mutations, differ among cancers of different tissue origin (Hollstein *et al.*, 1991). In hepatocellular carcinoma (HCC) mutation within the p53 gene occurs mainly at codon 249 (Bressac *et al.*, 1991, Hsu *et al.*, 1991).

Hepatocellular carcinoma is one of the most frequently occurring cancers worldwide and high incidence areas are South-East Asia and sub-Saharan Africa. In these countries, hepatitis B virus infection and exposure to aflatoxin are the two major risk factors (Munoz & Bosch, 1987). It has been suggested that aflatoxin B1 is responsible for mutation observed in codon 249 of the p53 gene (Bressac *et al.*, 1991; Hsu *et al.*, 1991; Hayward *et al.*, 1991). Accordingly, the frequency of this mutation vary greatly depending on the country of origin of cancer patients (Bressac *et al.*, 1991; Hayward *et al.*, 1991; Ozturk *et al.*, 1991), and it has been linked to the degree of exposure to aflatoxin (Ozturk *et al.*, 1991). Point mutation in codon 249 of the p53 gene results in the abolition of a restriction site for the enzyme Hae III (Bressac *et al.*, 1991). Thus mutation screening could be performed, combining the polymerase chain reaction (PCR) with restriction analysis of the amplified DNA sequence.

As Senegal is a country where HCC incidence is one of the highest in the world (Diop *et al.*, 1981) and where people are highly exposed to aflatoxin (Wild *et al.*, 1990), we screened 15 HCC DNA samples from this country for mutation at codon 249 of the p53 gene.

### Materials and methods

Frozen liver specimens obtained at necropsy were collected from 15 Senegalese patients suffering from liver cancer. Tissues were frozen immediately after necropsy and stored at – 20°C until analysis. DNA was extracted from the liver by Dyke's procedure (1988) with minor modifications. Briefly, 50 to 100 mg of tumoral and non-tumoral tissue sections from the same patients were minced in 1 ml of PBS and incubated at 55°C for 2 h with 1 ml of a lysis solution which contained 0.4 M NaCl, 4 mM EDTA (pH 8), 3% SDS and 2.5 mg $ml^{-1}$ proteinase K (Boehringer Mannheim). Proteins were precipitated by addition of NaCl (at a final concentration of 1 M), shaking vigorously, followed by centrifugation at 2500 rpm for 15 min. Total DNA was precipitated overnight (– 20°C) with ethanol and redissolved in 50 µl of 10 mM tris-HCl (pH 7.5), 1 mM EDTA.

Correspondence: Pierre Coursaget, Laboratoire de Microbiologie, Faculté de Pharmacie, 2 bis boulevard Tonnellé, 37042 Tours Cedex, France.
Received 11 March 1992; and in revised form 11 January 1993.

### *HBV DNA analysis*

Selected oligonucleotide primers (Thiers *et al.*, 1988) were synthesised using a DNA synthesiser apparatus (Gene assembler plus, Pharmacia). The primers 5'-CATCTTCTT-GTTGGTTCTTCTG-3' (Position 429–450) and 5'-TTAGG-GTTTAAATGTATACCC-3' (Position 824–844) are located in the S gene region of HBV. The reaction mixture contained 2 units of Taq polymerase (Promega), 50 pM of each primer, 200 µM of each dNTP and 5 µl of extracted DNA. The samples were subjected to 35 cycles of amplification including denaturation at 94°C for 30 s, annealing of primers at 50°C for 30 s, and elongation at 75°C for 1 min. The amplified products were run through 1.6% agarose gels and stained with ethidium-bromide.

### *p53 gene analysis*

DNA extracted from the various tumoral and non-tumoral tissue sections of the liver from 15 Senegalese patients were used. The PCR procedure consisted of 35 cycles of 94°C (30 s), 60°C (30 s), and 75°C (1 min). The primers used: 5'-GTTGGCTCGACTGTACCAC-3' and 5'-CTGGAGTCT-TCCAGTGTGAT-3' were two of those described by Bressac B *et al.* (1991).

After purification and concentration on a centrifugal-driven ultrafiltration membrane (Ultrafree-MC filter unit, Millipore), PCR products were then restricted to 3 h 30 min at 37°C with 10 U, Hae III (Pharmacia) in a final volume of 10 µl. The resulting digests were run through 5% NuSieve® 3/1 agarose (FMC Corporation) gels and stained with ethidium-bromide.

Hae III digestion resulted in two fragments of 75 bp and 35 bp lengths. In the presence of a mutation in codon 249 only one 110 bp fragment is detected after Hae III digestion.

To verify that conservation of the 110 bp fragment after Hae III digestion was not due to the presence of inhibitors of the enzyme in the tumour tissue, two amplified P53 samples were sequenced by Sanger's method using Vent DNA polymerase (Biolab) (Sears *et al.*, 1992) and $^{33}$PdATP (Zagursky *et al.*, 1991). The same set of sense and antisense primers utilised for DNA amplification by PCR were used as sequencing primers.

### Results

The presence of HBV DNA (Figure 1, Table I) was detected by PCR in 13 (87%) of the patients. DNA extracted from non tumoral liver tissue from ten of the patients showed a non-mutated p53 genotype. Mutation at codon 249 of the p53 gene (Figure 2, Table I) was detected in ten of the 15

**Figure 1** Detection of HBV DNA by PCR in liver tissue from HCC patients. Lanes 2-6 show bands at 417 base pairs. Lane 7: negative control. Lane 1 and 8 marker fragments generated by Bgl 1 and Hinf 1 cleavage of phage pBR 328 DNA.

**Figure 2** Detection of mutation at codon 249 of the p53 gene by digestion of specific amplified sequences with Hae III. Lane 2 shows the p53 amplified sequence (110 bp) before restriction, lane 4 and 7 show bands at 35 and 75 base pairs indicating total cleavage of the original 110 base pair fragment, lane 3 and 6 show the mutated p53 gene, and lane 5 show presence of both wild type and mutated P53 gene. Lane 1 and 8 marker fragments generated by Hae III cleavage of phage ΦX 174 DNA.

tumours tested (67%). The mutation was observed in eight out 13(62%) of HBV DNA positive patients and in the two patients without evidence of HBV infection. Seven out of the ten tumours with codon 249 mutation also had wild-type 249 sequences (Figure 2). This could be due either to the presence of both mutant and wild type p53 gene alleles in the tumours or to the presence normal hepatocytes and/or blood cells in the samples tested. Amplified DNA from two mutated samples were sequenced and mutation AGG to AGT was observed at codon 249 in both.

### Discussion

The frequency of mutation within codon 249 of the p53 gene observed in Senegal (67%) is the highest reported to date and is higher than that observed by Ozturk *et al.* (1991) in Mozambique (53%) and by Hsu *et al.* (1991) in China

**Table I** HBV-DNA and P53 mutation detection in HCC patients

| | | *P53 mutation in codon 249* | |
|---|---|---|---|
| *Case No* | *HBV-DNA* | *Normal tissue* | *HCC tissue* |
| 1 | + | ND | + |
| 2 | + | – | + |
| 3 | + | ND | + |
| 4 | + | – | + |
| 5 | + | – | + |
| 6 | + | – | – |
| 7 | + | – | – |
| 8 | + | ND | + |
| 9 | – | – | + |
| 10 | – | ND | + |
| 11 | + | – | + |
| 12 | + | – | + |
| 13 | + | ND | – |
| 14 | + | – | – |
| 15 | + | – | – |

ND = not done.

(50%). It must be noted that in Asian countries with high economic standards and low levels of aflatoxin intake, such as Japan, Taiwan and South Korea, mutation at codon 249 has not been observed (Ozturk *et al.*, 1991; Murakami *et al.*, 1991; Hosono *et al.*, 1991), in contrast to regions with high aflatoxin intake like the Qidong province of China or Vietnam (Hsu *et al.*, 1991; Ozturk *et al.*, 1991). In Africa, where aflatoxin intake is very high, the two countries tested had a high frequency of P53 mutation at codon 249. The results obtained in Senegal confirm the association between high aflatoxin intake and high frequency of mutation in codon 249 of p53 gene in hepatocellular carcinomas.

Fujimoto *et al.* (1992) did not find this P53 mutation in four non-human primates having aflatoxin B1 induced primary hepatocellular carcinoma, and suggested that hepatitis B virus could be a prerequisite for aflatoxin to induce this mutation. In this study, we observed two human cases without evidence of current HBV replication but with P53 mutation at codon 249. However, all HBsAg negative liver cancer patients from Senegal we have tested (Coursaget *et al.*, 1985), have evidence of past HBV infection.

Factors associated with chronic inflammatory and hepatic regeneration changes induced by hepatitis B virus, have been found to be important risk factors for hepatocarcinogenesis. In addition, loss of normal p53 function due to mutations may be a key step during malignant transformation of hepatocytes. Harris (1991) suggested that the interaction of a virally encoded protein with the specific mutant p53 provides a growth advantage in hepatomas. This hypothesis has been confirmed by Sell *et al.* (1991) since hepatitis B virus acts synergistically with aflatoxin in transgenic mice to produce neoplasia of the liver.

### References

BRESSAC, B., KEW, M., WANDS, J. & OZTURK, M. (1991). Selective G to T mutations of p53 gene in hepatocellular carcinoma from South Africa. *Nature*, **350**, 429-431.

COURSAGET, P., YVONNET, B., BARRES, J.L., PERRIN, J., TORTEY, E., DIOP, B., KOCHELEFF, P. DUFLO, B., MBOUP, S., DIOP-MAR, I., BOCANDE, J.E. & CHIRON, J.P. (1985). Hepatitis B virus and primary liver cancer in Intertropical Africa. *Rev. Epidem. et Santé Publ.*, **33**, 267-275.

DIOP, B., DENIS, F., BARIN, F., PERRIN, J., CHIRON, J.P., GOUDEAU, A., COURSAGET, P. & MAUPAS, P. (1981). Epidemiology of primary hepatocellular carcinoma in Senegal. *Prog. Med. Virol.*, **27**, 35-40.

DYKES, D.D. (1988). The use of biotynilated DNA probes in parentage testing: non isotopic labelling and non-toxic extraction. *Electrophoresis*, **9**, 359-368.

FUJIMOTO, Y., HAMPTON, L.L., LUO, L.D., WIRTH, P.J. & THORGEISSON, S.S. (1992). Low frequency of P53 gene mutation in tumours induced by aflatoxin B1 in nonhuman primates. *Cancer Res.*, **52**, 1044-1046.

HARRIS, A.L. (1991). Telling changes of base. *Nature*, **350**, 377-378.

HAYWARD, N.K., WALKER, G.J., GRAHAM, W. & COOKSLEY, E. (1991). Hepatocellular carcinoma mutation. *Nature*, **352**, 764.

HOLLSTEIN, M., SIDRANSKY, D., VOGELSTEIN, B. & HARRIS, C.C. (1991). p53 mutations in human cancers. *Sciences*, **253**, 49-53.

HOSONO, S., LEE, C.S., CHOU, M.J., YANG, C.S. & SHIH, C. (1991). Molecular analysis of the p53 alleles in primary hepatocellular carcinomas and cell lines. *Oncogene*, **6**, 237-243.

HSU, I.C., METCALF, R.A., SUN, T., WELSH, J.A., WANG, N.J. & HARRIS, C.C. (1991). Mutational hotspot in the p53 gene in human hepatocellular carcinoma. *Nature*, **350**, 457-428.

OZTURK, M., BRESSAC, B., PUISIEUX, A., KEW, M., VOLKMANN, M., BOZCALL, S., BELLA MURA, J., DE LA MONTE, S., CARLSON, R., BLUM, H., WANDS, J., TAKAHASHI, H., VON WEIZSÄCKER, R., GALUN, E., KAR, S., CARR, B.I., SCHRÖDER, C.H., ERKEN, E., VARINLI, S., RUSTGI, V.K., PRAT, J., TODA, G., KOCH, H.K., LIANG, X.H., TANG, Z.Y., SHOUVAL, D., LEE, S.H., VYAS, G.N. & SAROSI, I. (1991). p53 mutation in hepatocellular carcinoma after aflatoxin exposure. *Lancet*, **338**, 1356-1359.

MUNOZ, N.M. & BOSCH, F.X. (1987). Epidemiology of hepatocellular carcinoma. In *Neoplasms of the Liver* Okuda, K. & Ishak, K.G. (eds), pp 3-19, Springer, Tokyo.

MURAKAMI, Y., HAYASHI, K., HIROHASHI, S. & SEKIYA, T. (1991). Aberrations of the tumor suppressor p53 and retinoblastoma genes in human hepatocellular carcinomas. *Cancer Res.*, **51**, 5520-5525.

SEARS, L.E., MORAN, L.S., KISSINGER, C., CREASEY, T., O'KEEFE, H.P., ROSKEY, M., SUTHERLAND, E. & SLATKO, B.E. (1992). CircumVent thermal cycle sequencing and alternative manual and automated DNA sequencing protocols using the highly termostable $Vent_R$ ($exo^-$) DNA polymerase. *Biotechniques*, **13**, 626-633.

SELL, S., HUNT, J.M., DUNSFORD, H.A. & CHISARI, F.V. (1991). Synergy between hepatitis B virus expression and chemical hepatocarcinogens in transgenic mice. *Cancer Res.*, **51**, 1278-1285.

THIERS, V., NAKAJIMA, E., KREMSDORF, D., MACK, D., SCHELLEKENS, H., DRISS, F., GOUDEAU, A., WANDS, J., SNINSKY, J., TIOLLAIS, P. & BRECHOT, C. (1988). Transmission of hepatitis B from hepatitis-B-seronegative subjects. *Lancet*, **i**, 1273-1276.

WILD, C.P., JIANG, Y.Z., ALLEN, S.J., JANSEN, L.A.M., HALL, A.J. & MONTESANO, R. (1990). Aflatoxin-albumin abducts in human sera from different regions of the world. *Carcinogenesis*, **11**, 2271-2274.

ZAGURSKY, R.J., CONWAY, P.S. & KASHDAN, M.A. (1991). Use of $^{33}P$ for Sanger DNA sequencing. *Biotechniques*, **11**, 36-38.